# Effect of Gold Nanoparticle Radiosensitization on DNA Damage Using a Quartz Tuning Fork Sensor

**DOI:** 10.3390/mi14101963

**Published:** 2023-10-21

**Authors:** Nadyah Alanazi, Reem Alanazi, Mahmoud Algawati, Khaled Alzahrani, Abdullah N. Alodhayb

**Affiliations:** 1 Department of Physics and Astronomy, College of Science, King Saud University, Riyadh 11451, Saudi Arabia; nalenazi@ksu.edu.sa (N.A.); renazi@ksu.edu.sa (R.A.); malgawati@ksu.edu.sa (M.A.); 2 Department of Clinical Laboratory Sciences, College of Applied Medical Sciences, King Saud University, Riyadh 12372, Saudi Arabia

**Keywords:** biosensor, radiosensitizer, gold nanoparticle, radiotherapy, DNA damage, DNA repair, quartz tuning fork, DNA structure

## Abstract

The development of sensor technology enables the creation of DNA-based biosensors for biomedical applications. Herein, a quartz tuning fork (QTF) sensing system was employed as a transducer for biomedical applications to address indirect DNA damage associated with gold nanoparticles (GNPs) and enhance the effectiveness of low-dose gamma radiation in radiation therapy. The experiment included two stages, namely during and after irradiation exposure; shift frequencies (Δ*f*) were measured for 20 min in each stage. During the irradiation stage, the QTF response to DNA damage was investigated in a deionized aqueous solution with and without 100 nm GNPs at different concentrations (5, 10, 15, and 20 µg/mL). Upon exposure to gamma radiation for 20 min at a dose rate of 2.4 µGy/min, the ratio of Δ*f*/Δ*T* indicates increased fork displacement frequencies with or without GNPs. Additionally, DNA damage associated with high and low GNP concentrations was evaluated using the change in the resonance frequency of the QTF. The results indicate that GNPs at 15 and 10 µg/mL were associated with high damage-enhancement ratios, while saturation occurred at 20 µg/mL. At 15 µg/mL, significant radiotherapy enhancement occurred compared to that at 10 µg/mL at 10 min after exposure. In the post-irradiation stage, the frequency considerably differed between 15 and 10 µg/mL. Finally, these results significantly depart from the experimental predictions in the post-radiation stage. They exhibited no appreciable direct effect on DNA repair owing to the absence of an environment that promotes DNA repair following irradiation. However, these findings demonstrate the potential of enhancing damage by combining GNP-mediated radiation sensitization and biosensor technology. Thus, QTF is recommended as a reliable measure of DNA damage to investigate the dose enhancement effect at various GNP concentrations.

## 1. Introduction

Radiotherapy (RT) is a highly effective treatment for cancer, with >50% of patients receiving it for curative or palliative purposes. This involves administering lethal doses of ionizing radiation (IR) to the tumor via either an external beam (conventional RT) or an internally implanted radiation source (brachytherapy). While technological advancements, including intensity-modulated and image-guided RT, can reduce the risk of side effects, they are often expensive and used in combination with other treatments for better results [1]. To overcome this challenge, metal nanoparticles (NPs) are often incorporated into tumor tissue or cells before irradiation to selectively enhance their radiation sensitivity using heavy-element contrast agents, which exhibit high-energy absorption coefficients, leading to a considerable increase in the dose deposited in their vicinity [2]. Studies have reported that NPs, specifically gold NPs (GNPs), can enhance the efficacy of radiation treatment. Berbeco et al. investigated the effect of GNPs on cell damage using a clinical 6 MV beam at different depths and reported a significant increase in DNA damage caused by GNPs, suggesting new possibilities for GNP-aided radiation therapy [3]. Furthermore, Shahhoseini et al. reported that the dose enhancement factor (DEF) for cancer cell lines with 15 nm silver NPs (AuNPs) at 1 mM (2% *w*/*w*) was ~1.6 [4]. In addition, Cho et al. demonstrated that in tumors administered with 30 mg/g AuNPs, the DEF was ~1.3, as revealed by Monte Carlo [5]. Furthermore, Siam et al. [6] developed a population model for double-strand breaks (DSBs) and misrepaired cells following IR to establish a mathematical relationship for the interactions among IR, radiosensitizer, and the dose deposited by the radiosensitizer.

Su et al. also investigated the use of quartz tuning forks (QTFs) for biosensor applications. Biosensor function is achieved by coating the tuning fork surfaces with specific biomolecules and measuring the subsequent mass loading due to the binding of complementary analytes [7]. In addition, Alanazi et al. used a QTF sensor to detect low doses of gamma radiation with a fast response time. Three types of QTFs were used, including uncoated and gold-coated versions, and increasing the surface area of the gold coating substantially enhanced the radiation sensitivity [8]. Furthermore, Demir et al. used QTF as a chemical or physical sensor employing melanin NPs to create a target-specific mass-sensitive biosensor and modified it for the first time with MNP [9]. Shimoda et al. [10] investigated the frequency shift of a quartz oscillator due to gamma and beta radiation.

Particular interest has been focused on GNPs owing to their high atomic number, small size, natural tendency to accumulate in tumors, biocompatibility, low toxicity, relatively easy synthesis, and ability to bind to functional moieties within a biological target. Their versatility renders them highly desirable in numerous applications, including targeted drug delivery and radiotherapy [11,12]. Reportedly, materials with high atomic numbers (e.g., gold: Z = 79) absorb more energy when irradiated than materials with low atomic numbers. This local absorption triggers the emission of low-energy secondary electrons from the material that directly damage DNA while inducing indirect damage. This further generates microscale ionization clouds that cause water radiolysis and reactive oxygen species (ROS) production concentrated around the sites where the NPs are located. Consequently, irradiation amplifies the radiation dose within limited cell volumes, resulting in greater radiation damage and more efficient tumor cell killing. Additionally, NPs can generate ROS on their own, even in the absence of radiation, which is reportedly associated with their cytotoxicity. In addition to DNA damage, an increased level of ROS can lead to reaction with biomolecules, triggering cell death through numerous mechanisms [13]. The increase in the effect of a dose when it is delivered in the presence of GNPs is known as the “sensitization enhancement ratio” (SER). Therefore, if GNPs can accumulate in specific tissues, this would open the door to differential enhancement in tumoral tissues, allowing lower radiation doses to achieve adequate effects. However, it has been observed that GNPs induce higher SER than expected only owing to their physical conditions. Furthermore, the radiosensitizing effect of GNPs is partially triggered by increased ROS production compared to cells irradiated in the absence of GNPs. ROS can react with DNA, inducing DSBs and affecting cell viability. Moreover, without irradiation, GNPs increase oxidative stress in cells by interfering in the activity of some antioxidant enzymes. Because ROS have a limited lifespan, it seems reasonable that the presence of GNPs within cells would exhibit a greater radiosensitizing effect [14].

Herein, QTFs were used as biosensors, especially as a biological discriminator of the extent of DNA damage during radiation and the presence or absence of NPs. Biosensors possess a vast range of applications, encompassing healthcare, point-of-care testing, drug discovery, environmental monitoring, differential gene expression monitoring, forensic analysis, biodefense, and bioresearch. One characteristic of resonators developed using tuning fork frequencies is their wide application in biomedical research, including biosensor technology studies, which has attracted considerable attention [15,16]. In this regard, QTFs have emerged as a potent tool for biosensing applications [16]. The proof-of-principle of such sensors involves mechanically actuating tuning forks, either through an additional piezoelectric element placed at the bottom of the device or self-dispensing via tuning fork electrodes. The underlying principle of such sensors is that increased mass loading due to the adsorption of certain biomolecules onto tuning fork surfaces can change the measured frequency response [17].

This study aimed to assess how 100 nm spherical GNPs at varying concentrations can enhance radiation sensitivity by interacting with DNA in deionized water in the presence and absence of GNPs. We investigated ROS generation in the presence of GNPs using a sensor sensitive to higher-frequency deflection as an indicator of dose elevation and ROS free radical generation in indirect gamma reactions. In general, DNA damage was measured by revisiting the use of the event scoring function via the QTF technique. The experiments were conducted in three phases: an initialization stage involving setting up the experiment; an irradiation stage wherein the DNA damage was measured using the function of recording the frequency shift using the fork (the same calculation was conducted for the samples prepared with and without NPs); and the final stage involved DNA reconstruction after exposure and a discussion of the causes of DNA repair. In all the stages, a comparison of the deflection results of the fork frequencies was presented as a function of time and concentration. The radiosensitization of DNA is mediated by NPs at different concentrations. Therefore, we hypothesized that damage enhancement would increase with increasing concentration and time and that this enhanced damage could reduce the dose of therapeutic radiation in the presence of GNPs compared to that when only radiation is applied, which can help protect normal tissues from damage. Thus, the concentration of GNPs necessary to produce significant dose enhancement must be reduced for clinical applications.

## 2. Materials and Methods

### 2.1. Materials

**Figure 1:**
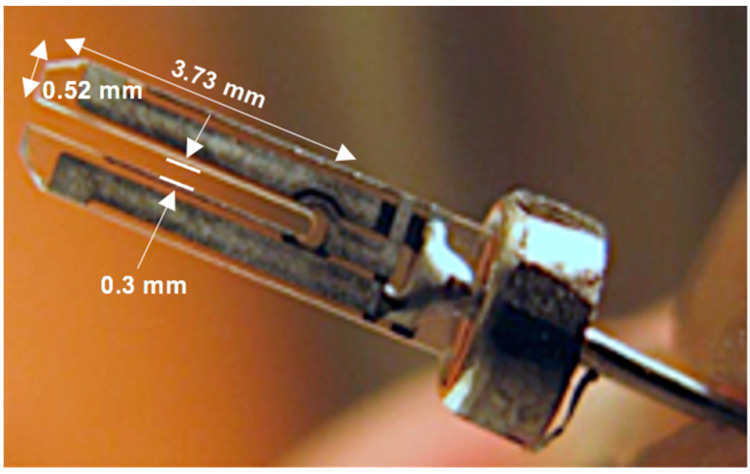
The QTF sensor used in this study.

A spherical gold nanoparticle with a diameter of 100 nm and concentration of 1 mg/mL in water was purchased from the NanoComposix Company (San Diego, CA, USA) and diluted in DI water at different concentrations (5, 10, 15, 20 µg/mL). Commercial genomic DNA with a concentration of 2 ng/µL was purchased from the Applied Biosystems Company, and DI water and quartz tuning forks were purchased from the FOURIEN company (Edmonton, AB, Canada). The tuning fork is shown in Figure 1.

### 2.2. Samples Preparation

**Figure 2:**
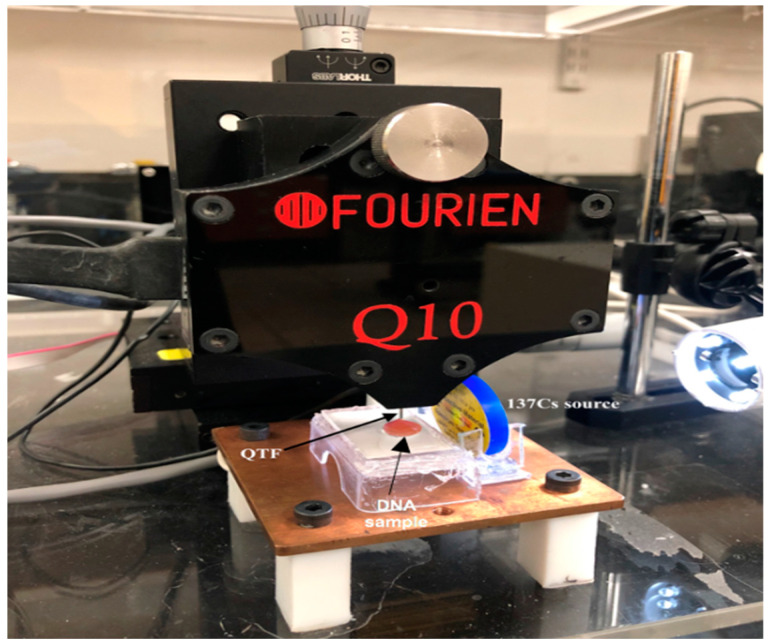
Experimental geometry setup: the QTF appears perpendicular to the sample, with the source located 1 cm away from the sample.

The DNA–gold nanoparticle solution samples were prepared by mixing 2 ng/µL DNA with gold nanoparticles suspended in water at different concentrations (5, 10, 15, and 20 µg/mL) with a volume ratio of 1:1. To observe the changes in the DNA samples caused by radiation in the presence of gold nanoparticles, the forks were immersed in 60 µL drops of the DNA–gold nanoparticle solution at different specific concentrations of gold nanoparticles, as illustrated in Figure 2. The fork demonstrates the DNA alterations with the variation in its resonance frequency.

### 2.3. Measurement Experiments

The measurements were carried out using a QTF measurement device, Quester Q10 (Fourien, Edmonton, AB, Canada). This instrument comprises a QTF as a piezoelectric transducer, which is designed as a mechanical resonator. The system is thoroughly explained elsewhere in [18]. The fork is highly symmetrical and mechanically coupled to reduce the damping losses and achieve a high-quality factor. The effect of radiation on the DNA nanoparticles in the presence and absence of gold nanoparticles was investigated by measuring the variation in the resonance frequency of the QTF submerged in the DNA solution. To measure the resonance frequency of the QTF, a signal was swept from 31 to 35 kHz. The resonance frequency of the QTF during and after radiation was continuously measured by Quester 10 software. Then, the resonance frequency was determined periodically every 5 min.

### 2.4. Irradiation Process

The samples were exposed to the synthetic radioactive isotope cesium-137 (137Cs; half-life: 30.2 years, original activity: 5 μCi), which produces gamma rays with an energy of 662 keV at different time intervals (different doses) and at room temperature (25 °C). Specifically, the samples were directly (in a parallel direction) exposed to gamma radiation to ensure that the entire DNA sample in a deionized aqueous solution received radiation with and without the different concentrations of NPs. The dose was delivered uniformly to all the samples at a distance of ~1 cm from the source over 20 min.

## 3. Results

### 3.1. Irradiation Stage

**Figure 3:**
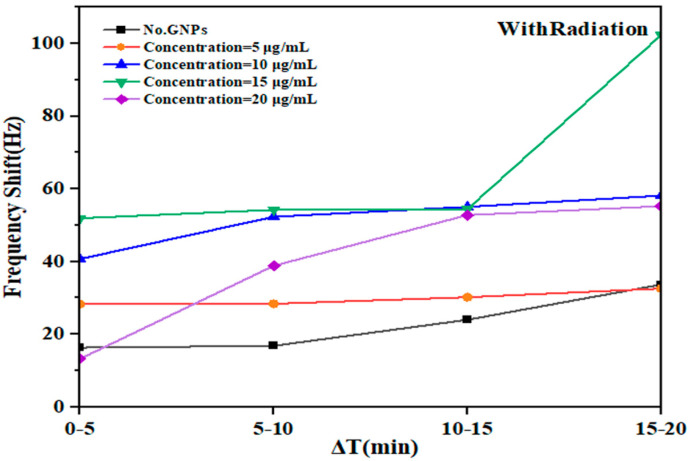
Fork response to DNA damage in deionized aqueous solution with and without GNPs at different concentrations following exposure to gamma radiation for 20 min where the ratio (Δf/ΔT) indicates the increased response of fork displacement frequencies obtained with and without GNPs.

During the irradiation phase, the Δ*f* test indicated the DNA damage in the presence of GNPs, and the results are shown in Figure 3. The radiation stage showed rapid direct DNA damage in the first 10 min of exposure, while the damage induced by irradiation peaked twice after irradiation for 10 min. The levels of DNA damage reached the ∆𝑓 maxima of 16.85, 28.3, 52.3, and 38.8 Hz at 0, 5, 10, 15, and 20 µg/mL per frequency, respectively, for each concentration 10 min after radiation. The DNA damage appeared to peak after 10 min of irradiation, with mean frequency numbers of 33, 32.6, 58.1, 102.2, and 55.26 Hz at 0, 5, 10, 15, and 20 µg/mL respectively, without and with GNPs, respectively. The damage induced by irradiation peaked at 10 min for both the applied GNP concentrations, and the presence of GNPs caused significant differences in the maximum average number of frequency deviations for all the concentrations in all cases, with the exception of 5 µm.

Therefore, based on the relationship of the DNA dose-response to damage at different GNP concentrations, which was determined as the shift frequency induced by the fork, there was a significant correlation between increasing the dose and shift frequencies during radiation (Table 1). There was a notable frequency shift in the radiation exposure region, indicating the extent of the response of the DNA to radiation (Figure 3). According to Table 1, the increase in the resonance frequency of the QTF was associated with an increase in the DNA damage. The frequency value considerably rose from 54.4 Hz to 102.2 Hz within an exposure time of 10 min.

**Figure 4:**
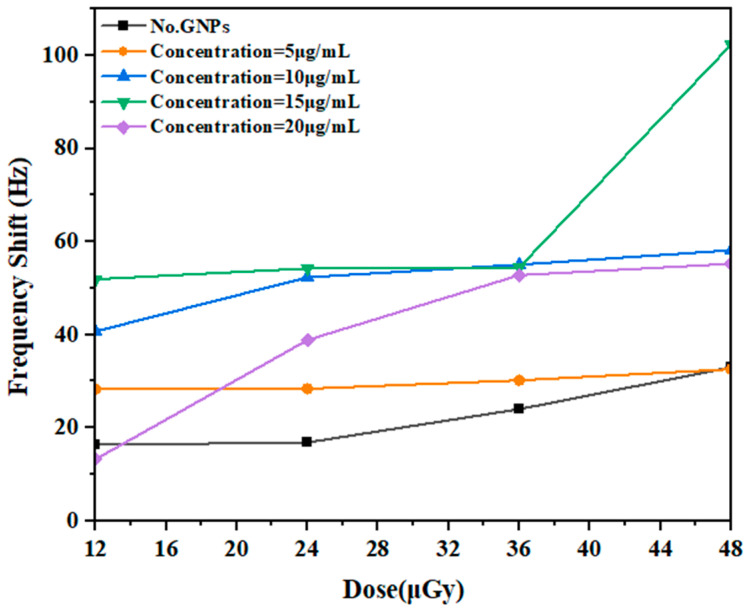
Frequency shift as a function of radiation dose irradiated with a 137Cs source in the presence of GNPs.

Specifically, herein, there was a significant increase in the DNA strand breaks at a GNP concentration of 15 µg/mL, which was associated with significant DNA damage. Furthermore, exposure to GNP concentrations of 10, 20, and 5 µg/mL induced less DNA damage, as indicated by the shift frequency ∆𝑓 of 102.3 Hz at an absorbed dose of 12 μGy during the initial 5 min exposure, as determined by measuring the fork shift frequencies. The dose’s levels continued to rise and reached 36 µGy, peaking at 48 μGy during exposure. The levels at 10 min of exposure were twice as high as those observed at the start of exposure with a GNP concentration of 15 µg/mL. Based on the dose–response relationship shown in Figure 4, this dose was selected as the maximum dose for the experiments. The frequency shift observed in the radiation-exposed region indicates the extent of the response of DNA to radiation, highlighting the importance of monitoring the effects of radiation on DNA.

**Figure 5:**
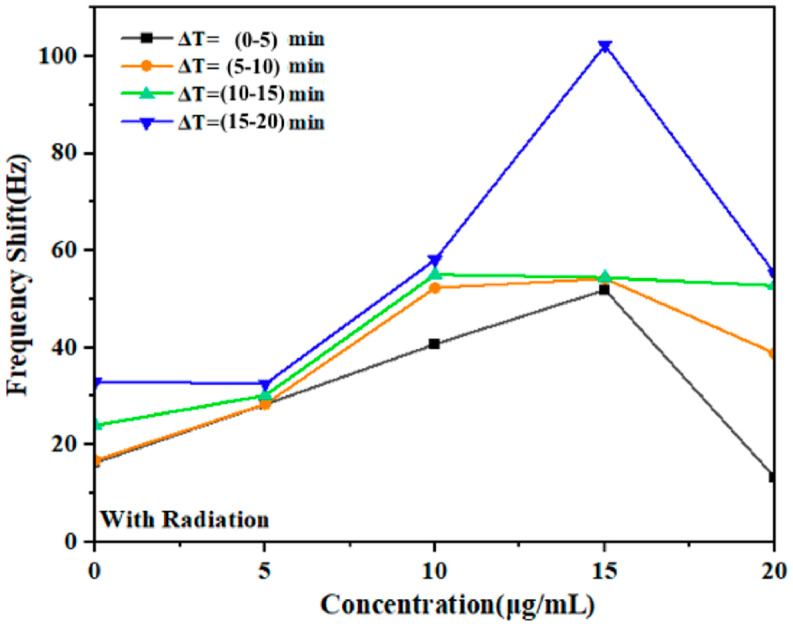
Effect of radiosensitization by GNPs at concentrations of 0, 5, 10, 15, and 20 µg/mL on the increase in DNA damage upon irradiation with gamma rays.

A similar pattern of the frequency change was observed in response to GNP concentrations of 15 and 10 µg/mL throughout the time course. As observed in Figure 5, the uptake of the GNPs was proportional to the damage concentrations. Au uptake increased with increasing concentrations of DNA in the pre-source condition, and the uptake of the GNPs differed for each analyzed concentration, in the following order: 20 > 10 > 5 > 15 µg/mL. For the GNP concentration of 5 µg/mL, no significant difference was observed in the frequency shift, regardless of the concentrations during the initial stage. Furthermore, statistically significant differences were observed at 10 and 15 µg/mL GNPs compared to higher GNP concentrations. However, the concentrations were similar during the first stage, with the highest concentrations at 5 µg/mL, followed by 10, 20, and 15 μg/mL in DNA.

Where the NPs exerted a radiosensitization effect on DNA damage caused by 137Cs radiation at low doses, the results of the radiation were EFs of 1.7–0.9, 2.4–1.7, 3.0–3.1, and 0.8–1.6 for 5, 10, 15, and 20 µg/mL, respectively (Table 2) after 20 min of exposure, of which the EF at 15 µg/mL was the largest because it was calculated from the damage yields with/without AuNPs. Our data regarding the improvement of bond-breaking productivity at concentrations of 10 and 15 µg/mL with high frequencies can be compared with the values obtained at concentrations of 20 and 5 µg/mL with low frequencies, using the same concentration of DNA in deionized aqueous solution and under the same experimental conditions. The reported EF values were 3.1 and 2.5 for bond breaking for 15 and 10 µg/mL, respectively, which indicates greater damaging power than the non-NP DNA concentrate used in this study. The results reveal that the GNPs that generated larger amounts of ROS at high-frequency deflection exhibited a greater dose-enhancing effect on DNA damage, as the frequency deflection did not differ between the concentrations of 10 and 15 µg/mL (Figure 5). The increase in bond breaking can be explained by the fact that dose enhancement by GNPs occurs owing to increased ROS generation by low-gamma-irradiated GNPs. Conversely, ROS production was lower at an NP concentration of 5 µg/mL at the same dose. Note that there was a decrease in the ROS yield at a concentration of 20 µg/mL, indicating lower ROS production, presumably due to GNP aggregation. The inset of Figure 5 shows the linear relationship between the dose from 12 to 48 µGy and the resonance frequency shift with different concentrations of GNPs. This indicates very high sensitivity and linearity, especially at 15 µg/mL. Thus, the QTFs can be deployed as radiation dosimeters in low-dose applications.

In terms of enhancement, a strongly significant increase in enhancement was demonstrated with all concentrations of NPs, which correlated with significant changes in the formation of strand breaks. Figure 4 and Figure 5 show DNA damage as a function of the doses delivered with different concentrations of 100-nm GNPs. It decreased at a concentration of 5 μg/mL with the dose in the irradiation stage, indicating that the irradiation increased the incidence of strand breaks with increasing concentration, while the decrease at the 5 μg/mL concentration was much higher than that observed in the pre-irradiation stage. Conversely, the decreases in the concentration to 10 and 15 μg/mL were not significantly different compared with the findings in the irradiation stage, where the extent of the DNA damage increased with increasing doses in both concentrations, indicating an increase in strand breaks due to gamma-ray irradiation. Furthermore, no significant changes were observed in the sample at a concentration of 20 μg/mL. In summary, the DNA damage increased with time, which was calculated as EF values, for GNPs at the measured concentrations. These values were compared with those in the post-irradiation phase. The EFs were also obtained for the GNPs relative to the control over time.

**Figure 6:**
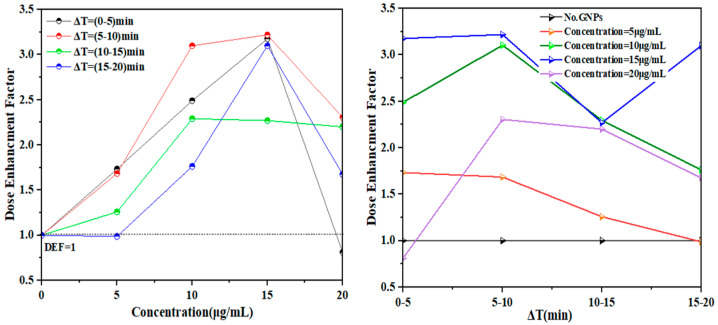
Frequency shift of the fork observed in the DNA in deionized water with GNPs at particular concentrations following exposure to radiation. Dose enhancement factor as a function of GNP concentration (5, 10, 15, 20 µg/mL) (**left**), and with different exposure times (**right**).

From the deposited gamma dose in the samples indicated by the frequency shift of the fork, the EFs were determined for the DNA in deionized aqueous solution with and without GNPs at different concentrations (Figure 6). The EF was measured relative to the dose deposited in the damaged region, and it is indicated by the shifts in the fork frequencies generated from the area of the quartz crystal and the inclusion of the shifts of the measured frequencies. The exact values of these parameters are provided in Table 2. The results of the damage enhancement by modality are presented below the EF and were determined according to Equation (1). We plotted the EF values against the different concentrations of GNPs to determine the extent to which the GNPs enhanced the DNA damage. An EF of >1 indicates that the concentration of GNPs increased the level of DNA damage, while an EF of <1 indicates no DNA damage. The formula for calculating the EF features the level of DNA damage with GNPs divided by the level of DNA damage without GNPs, as shown in Equation (1):

(1) $$EF=\frac{{\left(\Delta f\right)}_{DNA\;damage\;with\;GNPs}}{{\left(\Delta f\right)}_{DNA\;damage\;without\;GNPs}}$$

In general, this value (here EF = 1–3.2) had to be determined experimentally for each concentration during the experiment by solving Equation (1) for EF. According to our experience, the EF ranges between 1 and 3.2 depending on the concentration of NPs in the samples. Note that the EF for a pure acid in deionized water solution can differ mainly according to the concentration of NPs. However, the NP-specific EF for any given NP concentration can be readily determined using a QTF according to the shift frequency during the radiation stage. Finally, the saturation was observed in the absorbance at a concentration of 20 μg/mL in DNA, but not when the DNA was treated in an aqueous solution with GNPs at the lowest concentrations. The concentration of NPs mixed with DNA and water was greater than that of Au for all concentration levels tested, indicating a higher overall shift frequency at a concentration of 15 μg/mL compared to that at a concentration of 10 μg/mL. This trend was also observed for 20 and 5 μg/mL, especially at low concentrations. When comparing different concentrations in the DNA, a considerably higher shift frequency appeared for a concentration of 15 μg/mL. In the case of exposure to gamma radiation, the (EF) was comparable between the concentrations of 20 µg/mL and 10 µg/mL, but not for the 15 µg/mL concentration, where the frequency deviation was approximately three times higher than at 5 µg/mL after a 20 min exposure.

### 3.2. Post-Irradiation Stage

**Figure 7:**
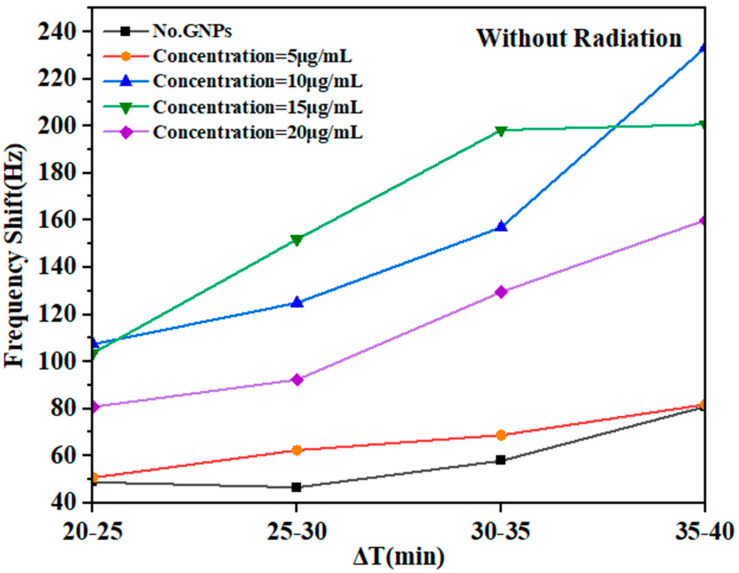
Fork response to DNA damage in deionized aqueous solution with and without GNPs at different concentrations, where the ratio (Δf/ΔT) indicates nonconjugation of the DNA and severe DNA damage at 20 min after exposure.

After the irradiation stage, there was no significant difference in the concentration as a function of time (Figure 7), when the GNPs were used to assess the damage enhancement due to their concentration. The same samples with different concentrations of gold NPs (GNPs) (0, 5, 10, 15, and 20) µg/mL with DNA in an aqueous solution were used to investigate the effect of the amount of GNPs on DNA damage after irradiation, and the shift frequencies were measured for each of these samples and compared to the results obtained during the irradiation stage. The observed data were analyzed to determine the role of DNA repair in the presence of GNPs. The frequency shifts ∆𝑓 ~ 48.84–80.92 Hz, ∆𝑓 ~ 50.75–81 Hz, ∆𝑓 ~ 107.39–232.73 Hz, ∆𝑓 ~ 103.6–200.71 Hz, and ∆𝑓 ~ 80.85–160.06 Hz were measured by fork shift for the concentrations of 0, 5, 10, 15, and 20 µg/mL, respectively. These results are summarized in Table 3.

Figure 7 plots the exposure–response curve of frequency shifts with GNPs for different concentrations. The semi-straight lines through the points for the GNPs with DNA in deionized aqueous solution indicate that the induced damage was linear with time up to approximately ∆𝑓 ~ 200 Hz at a concentration of 15 µg/mL. DNA damage was also observed in an aqueous solution at other concentrations (Table 1). The curves in Figure 7 demonstrate the dependence of the frequency shift (Δf) of the fork as well as the DNA damage in the recovered samples as a function of post-irradiation exposure. However, the linear region after exposure increased because of the lack of a suitable environment.

**Figure 8:**
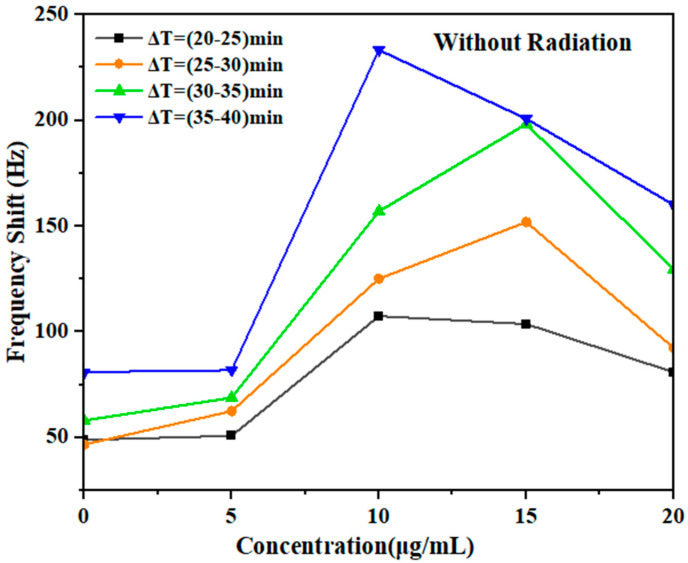
Frequency shift distribution of each sample according to the concentration of NPs with DNA in aqueous solution post-irradiation as a function of time. The histogram shows the findings after irradiation.

The results show a rise in the frequencies of the QTF immediately following exposure; then, the frequency shift increased at a concentration of 15 µg/mL (Table 3). The damage was estimated immediately and after irradiation (20 min) for each a period of damage and plotted against the post-irradiation time. (Figure 8). The high frequencies of indirect repair reactions observed suggest that the gamma radiation caused extensive damage to the DNA, which is challenging to repair through recombination due to the absence of a suitable environment. Consequently, the function of DNA damage response proteins, such as gH2AX, ATM, 53BP1, RAD51, and the MRE11/RAD50/NBS1 complex, at the DNA damage sites could have significant implications for interpreting the observed changes in recombination-related genes located near the DNA double-strand breaks, contributing to their repair. While many studies have shown that damage sites are processed by recombination or base excision repair enzymes in living cells, a certain fraction of the damage remains persistent and can lead to serious genetic effects.

## 4. Discussion

GNP treatment alongside radiation increases the extent of DNA damage compared to that induced by radiation alone. This demonstrates that the introduction of GNPs substantially impacts the response to DNA damage. By specifically targeting the concentration of GNPs in DNA with water at low-energy gamma rays, indirect irradiation damage can be reduced, allowing for more focused investigation into the impact of DNA damage.

Several studies have hypothesized that GNPs would increase DNA damage upon exposure to gamma rays, and the extent of this enhancement would depend on the concentration and exposure time. The results of this study are consistent with the those of numerous studies regarding the enhancement of GNPs with radiation, such as the study by Geng et al. [19]. When used together with IR, GNPs increase radiosensitization by promoting free radical production. Studies have revealed increased radical production when glucose-capped GNPs were present during irradiation using 90 kVp and 6 MV X-rays. Additionally, Misawa et al. reported that when GNPs in water were exposed to 100 kVp X-rays, hydroxyl radical (1.46-fold) and superoxide anion (7.68-fold) levels increased, which damage DNA [20]. Furthermore, the results of a study presented in [3] demonstrated the measurement of relative damage enhancement in the presence and absence of 50 nm GNPs during the irradiation of HeLa cells at depths ranging from 1.5 to 20 cm. The study revealed that there were relative increases in the promotion of DNA damage with the assistance of gold nanoparticles as the depth of radiation therapy increased. In addition, according to the results of Burn et al., there is a relationship between the AuNP size and the DEF for single-thread spacers (SSBs). As the GNP size increased in the range of 8–92 nm in water, the DEF of the SSBs increased from 1.2 to 3.0 [21].

In a study by Wang et al., GNPs were used to functionalize quartz crystal microbalance (QCM) for detecting Escherichia coli DNA; GNPs of two different sizes were used to increase the sensitivity. This was due to the sensitivity of the QCM sensors and the amplification of the signal that includes the NPs. The NPs were effective in detecting DNA in QCM because they had a relatively large mass compared to the bulk of the DNA molecules, and the NPs operated as “mass enhancers” and signal amplifiers, thus extending the limits of QCM DNA detection [22]. In addition, Chen et al. developed a QCM DNA sensor for detecting foodborne pathogens using NP amplification. They used a sandwich hybridization approach, where one probe specific to *E. coli* O157:H7 was immobilized onto the QCM surface and a second probe was conjugated to GNPs, acting as a “mass enhancer” and “sequence verifier”. By amplifying the frequency change of the piezoelectric part, the oscillation frequency of the piezoelectric sensor decreased as the weight on the surface of the sensor increased [23]. Herein, the response of various concentrations of GNPs (5, 10, 15, and 20 μg/mL) mixed with DNA to low-energy gamma rays of Cs-137 in deionized aqueous medium was examined. The study evaluated the effects by measuring the frequency shift of the QTF (Figure 3). The experiments were conducted for 60 min, with each stage lasting 20 min. The mean values of the shift frequency were recorded every 5 min for each concentration level during irradiation exposure and after irradiation. The results reveal the response of the DNA-immersed QTF fork to different NP concentrations. The time courses of resonant frequency changes on the QTF sensor were compared among the GNP concentrations. A constant, linear frequency increase (associated with an increase in mass) was recorded when the sensor was exposed to GNPs. The slope of the linear increase (Δf/Δt) was proportional to the concentration of GNPs over time (Figure 3 and Figure 5). In addition, the findings revealed that DNA damage increased significantly after the initial 5 min of exposure, reaching 12 μGy depending on the concentration. The shift frequency (Δf) values at the concentrations 0, 5, 10, 15, and 20 µg/mL were also recorded as ∆𝑓 16.3, 28.3, 40.7, 51.9, and 13.2 Hz, respectively. Following a 10 min exposure, which reached 24 µGy, the DNA damage increased dramatically during a subsequent 20 min exposure, reaching 48 µGy. This resulted in a significant increase in damage at Δ𝑓 frequencies of approximately 33, 32.5, 58.1, 102.2, and 55.2 Hz, respectively (Figure 3). In addition, in the post-irradiation phase, the frequency significantly increased (Figure 7).

We also observed that the most reliable indicator of the sensor response to varying concentrations was obtained when the EF was determined in the presence of NPs. The EF was obtained by directly dividing the measurement in the presence of NPs by that in the absence of NPs during the exposure, within a range of EF ~ 1–3.2. As a result, we evaluated the specific EF as the response signal to DNA damage with GNPs as a sensor (Figure 6). This resulted in the highest enhancement damage. We found that calculating the EF for different concentrations was necessary to arrive at the linearly increased figure of the sensor response at a given concentration. Our experience indicates that a concentration of 15 µg/mL is the best improvement coefficient within limits ~ EF = 3.2.

Although significant changes in frequency were observed in the samples, they were lower than those observed specifically at concentrations of 5 and 20 µg/mL. This can be explained by comparing the findings at a high concentration of NPs, which indicates that accumulation may reach saturation at 20 µg/mL, and there is an increase in the production of ROS. The generation of ROS during the irradiation process increased the DNA damage because of increased exposure. The damage amelioration factor, which determines the shift in the resonance frequency of the QTF sensor, had concentrations of 10 and 15 µg/mL, which are associated with significant increases in the DNA damage compared to that induced by GNPs alone, whereas concentrations of 5 and 20 µg/mL exhibited no significant effect.

One of the important findings of our work is the increase in the resonance frequency of the QTF following the post-irradiation stage of the samples. This increase indicates DNA unpairing and severe DNA damage after exposure. At concentrations of 5, 10, 15, and 20 µg/mL, with the absence of GNPs, the frequency values were high (Δ*f* = 81.81, 233.3, 200.70, 160.05, and 80.92 Hz). In summary, gamma radiation leads to extensive DNA damage that is challenging to repair due to the absence of a suitable environment. The observed increase in the shift frequencies of indirect repair reactions suggests that gamma radiation causes substantial DNA damage, making reconstruction difficult. Numerous studies have demonstrated that although a damaged site can undergo processing via recombination through BER enzymes in living cells, a portion of the damage can persist, leading to substantial genetic effects, including mutation induction [24]. To investigate DNA damage response and repair under ex vivo conditions, we conducted experiments in an aqueous medium with varying GNPs concentrations. After irradiation under the same conditions, an observed increase in frequency indicated damage without subsequent repair. Although our results show that that radiation during irradiation with GNPs caused DNA damage, we were not able to determine whether these changes could be completely repaired due to the absence of an appropriate environment. Future investigations should establish conditions for DNA self-restoration after radiation exposure [25].

Finally, based on the trends observed in this study, we anticipate that the strategy of utilizing gamma radiation to enhance the concentration of NPs within DNA will significantly enhance the potential of radiotherapy. This in vitro study showed the relative change in DNA damage enhancement upon changing the treatment parameters using different GNP concentrations with and without irradiation. The clinical benefit of GNP-aided radiotherapy will also depend on the distribution and concentration of GNPs, in vivo cellular uptake, biological target, and subsequent physiological changes. Nonetheless, the results of this study are positive. With further accumulated effects, the clinical significance of this concept will be enhanced. Herein, we propose the use of GNPs with a shift in fork frequencies to assess the extent of DNA damage. One of the strategies that we suggest is to directly inject NPs into a DNA medium in a deionized aqueous solution and then submerge the fork substrates perpendicular to the DNA samples. We believe that this method can provide information for detecting the extent of DNA damage and determining the accumulation of NPs as a sensitive indicator of DNA damage by measuring the shift frequency of a fork. However, to ensure the effective treatment purpose, it is essential to consider the delivery of good efficacy at an appropriate concentration. A concentration of 20 μg/mL may not directly induce DNA damage; therefore, it is important to prevent the aggregation of NPs when administered at high concentrations.

## 5. Conclusions

This study demonstrates that the dose was increased by the presence of GNPs during irradiation, thereby causing an increase in the DNA damage. Such a finding was investigated using QTFs. The results of a two-stage experiment conducted during and after irradiation exposure independently mapped out the dependence on shift frequency, serving as an indicator of the radiosensitizing effects of GNPs on DNA in a deionized aqueous solution. The DNA damage in response to the absence and presence of GNPs at different concentrations (5, 10, 15, and 20 μg/mL) upon exposure to a 137Cs gamma radiation source for 20 min was evaluated. Additionally, the relative enhancement of the extent of DNA damage in the presence of GNPs compared to that in the absence of GNPs was confirmed using a shift frequency QTF sensor. Thus, the presence of GNPs significantly increased the extent of DNA damage. The extent of the DNA damage enhancement was directly proportional to the dose rate, with a range of 12–48 µGy being required to achieve improvement in the DNA damage 10 min after exposure at a dose of 24 µGy. Higher DNA damage enhancement was observed at a concentration of 15 μg/mL when considering the same NP among concentrations, with a maximum factor of 3.1 observed after 20 min of exposure. At the lowest concentration, 5 µg/mL, and the highest concentration, 20 µg/mL, we observe an almost negligible radiosensitizing effect. In contrast, at concentrations of 15 µg/mL and 10 µg/mL, the radiosensitization is clearly evident. The extent of the DNA damage enhancement was dependent on the ability of the GNPs to promote ROS generation, such as •OH radicals, with lower levels of ROS generated at 5 and 20 μg/mL, resulting in nonsignificant promotion of DNA damage enhancement, and DNA damage only occurred when the local concentration of GNPs was increased around the DNA.

This study found that following gamma-ray irradiation in the presence of GNPs, DNA damage increased and accumulated, ultimately resulting in complete DNA damage. The repair process was hindered owing to the absence of DNA damage response proteins in the medium, thereby rendering DNA repair difficult. These results suggest that using QTF alongside GNPs for radiotherapy would be appropriate for dose enhancement in biotechnological applications.

## Figures and Tables

**Table 1:** Frequency shift (Hz) due to exposure to gamma radiation at different concentrations measured every 5 min.

Concentration (µg/mL)	Time (min)			
	0–5	5–10	10–15	15–20
	Frequency Shift (Hz)			
No GNPs	16.34	16.85	23.99	33.00
5	28.33	28.39	30.17	32.59
10	40.70	52.33	55.06	58.14
15	51.91	54.24	54.43	102.29
20	13.26	38.84	52.80	55.26

**Table 2:** Relationships among the dose rate, enhancement factor (EF), and difference in the concentrations of GNPs according to shift frequencies of the fork measured every 5 min.

Concentrations (µg/mL)	Dose Rate			
	5 min	10 min	15 min	20 min
	12 µGy	24 µGy	36 µGy	48 µGy
	Enhancement Factor (EF)			
	0−5 min	5–10 min	10–15 min	15–20 min
No GNPs	1	1	1	1
5	1.733	1.685	1.257	0.987
10	2.490	3.105	2.295	1.761
15	3.177	3.219	2.269	3.099
20	0.811	2.305	2.201	1.674

**Table 3:** Frequency shift (Hz) without exposure to gamma radiation with different concentrations measured every 5 min.

Concentration (µg/mL)	Time (min)			
	20–25	25–30	30–35	35–40
	Frequency Shift (Hz)			
No GNPs	48.84	46.67	57.96	80.92
5	50.75	62.41	68.83	81.81
10	107.39	125.00	156.98	233.3
15	103.63	151.89	198.27	200.70
20	80.84	92.33	129.54	160.05

## Data Availability

All data generated or analyzed during this study are included in this article.
